# 3D Printed Conductive Multiscale Nerve Guidance Conduit with Hierarchical Fibers for Peripheral Nerve Regeneration

**DOI:** 10.1002/advs.202205744

**Published:** 2023-02-17

**Authors:** Yongcong Fang, Chengjin Wang, Zibo Liu, Jeonghoon Ko, Li Chen, Ting Zhang, Zhuo Xiong, Lei Zhang, Wei Sun

**Affiliations:** ^1^ Biomanufacturing Center Department of Mechanical Engineering Tsinghua University Beijing 100084 P. R. China; ^2^ Biomanufacturing and Rapid Forming Technology Key Laboratory of Beijing Beijing 100084 P. R. China; ^3^ “Biomanufacturing and Engineering Living Systems” Innovation International Talents Base (111 Base) Beijing 100084 P. R. China; ^4^ Department of Mechanical Engineering Drexel University Philadelphia PA 19104 USA

**Keywords:** 3D bioprinting, aligned microfiber, graphene oxide, nerve guidance conduits, nerve regeneration

## Abstract

Nerve guidance conduits (NGCs) have become a promising alternative for peripheral nerve regeneration; however, the outcome of nerve regeneration and functional recovery is greatly affected by the physical, chemical, and electrical properties of NGCs. In this study, a conductive multiscale filled NGC (MF‐NGC) consisting of electrospun poly(lactide‐co‐caprolactone) (PCL)/collagen nanofibers as the sheath, reduced graphene oxide /PCL microfibers as the backbone, and PCL microfibers as the internal structure for peripheral nerve regeneration is developed. The printed MF‐NGCs presented good permeability, mechanical stability, and electrical conductivity, which further promoted the elongation and growth of Schwann cells and neurite outgrowth of PC12 neuronal cells. Animal studies using a rat sciatic nerve injury model reveal that the MF‐NGCs promote neovascularization and M2 transition through the rapid recruitment of vascular cells and macrophages. Histological and functional assessments of the regenerated nerves confirm that the conductive MF‐NGCs significantly enhance peripheral nerve regeneration, as indicated by improved axon myelination, muscle weight increase, and sciatic nerve function index. This study demonstrates the feasibility of using 3D‐printed conductive MF‐NGCs with hierarchically oriented fibers as functional conduits that can significantly enhance peripheral nerve regeneration.

## Graphical abstract

A conductive multiscale nerve guidance conduit is employed in treating peripheral nerve injuries by integrating multiscale and multifunctional biomaterials with advanced 3D printing. The printed nerve guidance conduit promotes the growth of neuronal cells, facilitates neovascularization and M2 transition of macrophages, ultimately enhancing peripheral nerve regeneration and providing an innovative clinical treatment strategy from the tissue‐engineering perspective.

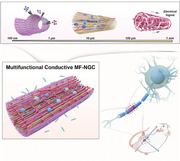

## 1 Introduction

Peripheral nerve injuries (PNIs) are one of the most common traumatic injuries to the nervous system and affect millions of people worldwide.^[^ ^1^ ^]^ Therefore, peripheral nerve regeneration has become a focus of significant clinical interest.^[^ ^2^ ^]^ In the clinic, autologous nerve grafts are commonly regarded as the gold standard treatment. Nevertheless, they have several limitations, including the shortage of donor nerves, mismatch in size, and donor site morbidity.^[^ ^3^ ^]^ In recent decades, significant research efforts have been invested in developing nerve guidance conduits (NGCs) as synthetic alternatives, providing temporary mechanical support and a favorable microenvironment for nerve regeneration.^[^ ^4^ ^]^ Although hollow NGCs were widely used in the early days of treatment with beneficial regeneration effects,^[^ ^5^ ^]^ these conduits are mostly ineffective in large lesions due to a lack of biophysical cues and mechanical stability.^[^ ^6^ ^]^ Topographical cues have been reported to induce a specific response in neuronal cells.^[^ ^7^ ^]^ For example, neurons grown on oriented microfibers or microgrooves prefer to extend neurites in a geometrically ordered pattern.^[^ ^8^ ^]^ Therefore, an intriguing trend concerns introducing anisotropic guiding cues into NGCs to enhance therapeutic effects in injured peripheral nerves.^[^ ^9^ ^]^

To date, several approaches have been developed to fabricate NGCs, including freeze‐drying,^[^ ^10^ ^]^ electrospinning,^[^ ^11^ ^]^ and 3D printing.^[^ ^12^ ^]^ A freeze‐drying technique using ice particulates as porogen materials can generate highly interconnected porous architectures within NGCs.^[^ ^13^ ^]^ Electrospinning is a popular and attractive technique for generating porous NGCs with ultrafine nanofibers mimicking the extracellular matrix of native tissue.^[^ ^14^ ^]^ However, the freeze‐drying technique has failed to produce NGCs with complex structures and regular pore shapes, whereas electrospinning has inherent limitations such as poor repeatability and customizability.^[^ ^15^ ^]^ 3D printing has become a promising alternative for creating NGCs with complex architectures; however, conventional extrusion‐based 3D printing techniques such as fused deposition modeling (FDM) typically produce relatively large fiber diameters (>100 µm), failing to provide directionality for regenerating axons.^[^ ^16^ ^]^ Recently, melt electrowriting (MEW) technology has gained increasing interest for its particular capability of producing fibers with diameters as small as a few micrometers while enabling structural complexity.^[^ ^17^ ^]^ To date, various NGCs have been produced with MEW printing, which offers superior control over fiber diameter and alignment.^[^ ^18^ ^]^ For example, Li et al. demonstrated that MEW‐printed poly(lactide‐co‐caprolactone) (PCL) fibers with a diameter of 10 µm, close to the size of cells, allow cells to sense and adhere to the fibers, thereby promoting cell elongation and orientation.^[^ ^19^ ^]^ However, they often lack mechanical stability under deformation due to the small size of MEW‐printed fibers.

Recently, researchers have begun to recognize the importance of the electroactive properties of NGCs, as they can transmit electricity or generate it upon external stimulation and activate critical intracellular signaling pathways.^[^ ^20^ ^]^ The transmission of bioelectrical signals is crucial to the functional restoration of peripheral nerves as neuronal cells are electroactive.^[^ ^21^ ^]^ Therefore, significant research has been invested in developing conductive NGCs by incorporating electroactive materials^[^ ^22^ ^]^ or surface coatings.^[^ ^23^ ^]^ For instance, carbon nanomaterials, including carbon nanotubes^[^ ^24^ ^]^ and graphene,^[^ ^25^ ^]^ have been confirmed to stimulate the proliferation and neurite outgrowth of nerve‐derived cells, including PC12 and Schwann cells; however, the poor dispersity of carbon nanomaterials severely compromises the printing capability of MEW and thus their conductivity.^[^ ^26^ ^]^ Conductive polymers such as polyaniline, polypyrrole, and polythiophenes have attracted particular attention for their easy‐to‐tailor properties and good processability^[^ ^27^ ^]^; however, significant variation in conductivity with these polymers, greatly affected by their structures and dopants, may pose potential challenges to their further application.

**Figure 1:**
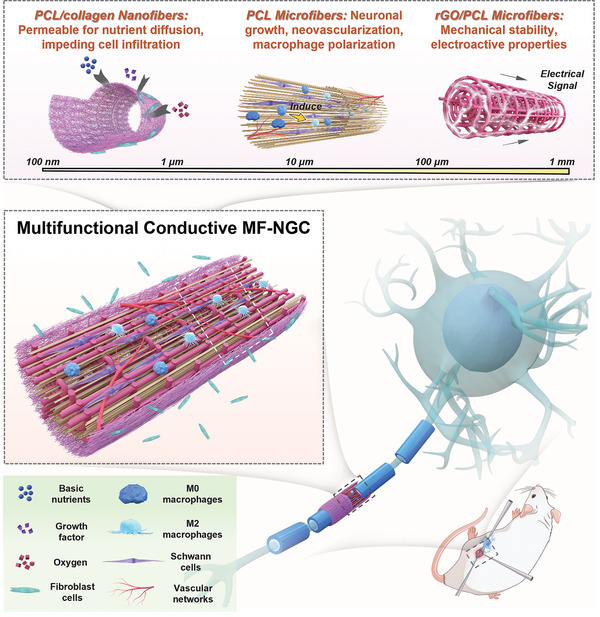
Overview of a multifunctional conductive MF‐NGC with hierarchical fibers for PNI treatment. The conductive MF‐NGC consists of hierarchical fibers with diameters ranging from 500 nm–120 µm, which were printed by MEW and electrospinning techniques. The multiscale fibers conferred multifunctionality to the MF‐NGCs as follows: i) The outer layer of the MF‐NGCs consisting of random PCL/collagen nanofibers (diameter: ≈500 nm), provides excellent permeability for nutrient diffusion and waste removal while impeding infiltration of fibrous scar tissue; ii) The internal layer of the MF‐NGCs mainly consisting of longitudinal PCL microfibers (diameter: ≈10 µm), provides anisotropic guidance for neuronal growth and the rapid recruitment of vascular cells and macrophages; iii) The intermediate layer of the MF‐NGCs consisting of rGO/PCL microfibers (diameter: ≈125 µm), provides the desired mechanical stability and electroactive properties to support neural growth.

**Figure 2:**
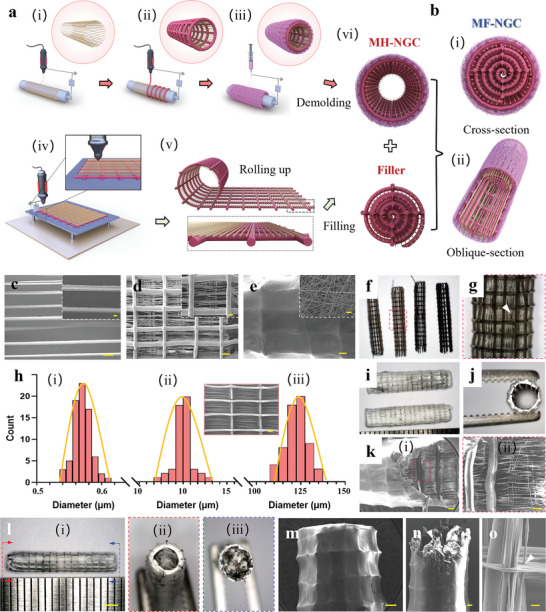
Fabrication and morphology of MF‐NGCs. a) Schematic illustration of the fabrication of MF‐NGCs, including i) MEW printing of PCL microfibers on a rotating mandrel; ii) MEW printing of rGO/PCL microfibers; iii) electrospinning of PCL/collagen nanofibers and removing from the mandrel to obtain an MH‐NGC; iv) MEW printing of a fibrous sheet consisting of PCL microfibers (yellow) and rGO/PCL microfibers (red); v) collecting and rolling up the fibrous sheet; and vi) filling the densely packed fibrous sheet into the lumen of MH‐NGC to obtain an MF‐NGC. b) Schematic illustration of a 3D printed MF‐NGCs. i) A cross‐section view of MF‐NGC; ii) an oblique‐section view of MF‐NGC. c) SEM images of MEW‐printed PCL microfibers. Scale bar, 100 µm (subset: 20 µm). d) SEM images of MEW‐printed rGO/PCL microfibers. Scale bar, 200 µm (subset: 100 µm). e) SEM images of electrospun PCL/collagen nanofibers. Scale bar, 200 µm (subset: 5 µm). f) Diameter distributions of hierarchical fibers within NGCs. Scale bar in (ii), 200 µm. g) Optical images of the conduits by alternative printing of PCL microfibers and rGO/PCL microfibers. Scale bar, 2 mm. h) A magnified view of the conduit in (g), marked by the red gridline. The white arrow indicates the white PCL microfibers. i) Optical image of MH‐NGCs in the top view. Scale bar, 2 mm. j) Optical image of MH‐NGCs at the cross section. k) SEM images of the semisection of the MF‐NGCs. ii) Magnified view of (i). Scale bar, 500 µm (subset: 200 µm). l) Optical images of MF‐NGCs in the top view. ii,iii) The cross‐section view at two ends of the MF‐NGCs marked in (i). Scale bar, 2 mm. m) SEM images of the exterior sheath of the MF‐NGCs. Scale bar, 500 µm. n) SEM images of the oblique section of the MF‐NGCs. The white arrow indicates the encapsulated microfibers with the conduit. Scale bar, 500 µm. o) SEM images of the grid patterns within the MF‐NGCs. The white arrow indicates longitudinal PCL microfibers with relatively smaller sizes. Scale bar, 50 µm.

To address these challenges, we proposed a conductive multiscale‐filled NGC (MF‐NGC) with hierarchical fibers with diameters ranging from 100 nm–125 µm, as illustrated in **Figure** **1**. The conductive MF‐NGCs are imparted with multifunctionality to enhance peripheral nerve regeneration as follows: i) at the nanoscale, random PCL/collagen nanofibers (diameter: ≈500 nm) were electrospun to generate the outer layer of MF‐NGCs, resulting in excellent permeability for nutrient diffusion and waste removal while impeding cell infiltration; ii) at the microscale, the longitudinal PCL microfibers (diameter: ≈10 µm) were printed by MEW, providing anisotropic guidance for neuronal growth; iii) at the mesoscale, the reduced graphene oxide (rGO)/PCL microfibers (diameter: ≈125 µm) were printed by MEW, providing the desired mechanical stability and electroactive properties to support neural growth. For this purpose, we developed a multiscale 3D printing platform by integrating MEW and electrospinning techniques. To achieve a conductive MF‐NGC, we first produced a conductive multiscale hollow NGC (MH‐NGC) by sequentially printing PCL microfibers, rGO/PCL microfibers, and PCL/collagen nanofibers on a rotating mandrel (**Figure** **2**a(i–iii)). Second, we fabricated fibrous sheets consisting of MEW‐printed PCL microfibers and rGO/PCL microfibers (Figure 2a(iv)), which were rolled up and filled into the lumen of MH‐NGCs (Figure 2a(v,vi)). We characterized the morphology, mechanical, and electrical properties of 3D‐printed conductive MF‐NGCs to determine their feasibility for peripheral nerve regeneration. We evaluated the effectiveness of hierarchical and anisotropic microfibers in guiding neurite growth by seeding PC12 neuronal cells and RSC96 Schwann cells on flat multiscale fibrous scaffolds. Last, we performed in vivo animal tests to assess the therapeutic efficacy of MF‐NGCs for peripheral nerve regeneration compared to MH‐NGCs and autografts.

## 2 Results and Discussion

### 2.1 Fabrication and Morphology of MF‐NGCs

Ideally, NGCs for peripheral nerve regeneration should possess the following key features: i) permitting the diffusion of growth factors to direct nerve regeneration toward the distal segment through diffusion gradients while impeding the infiltration of fibrous scar tissue; ii) providing the desired topographical cues to guide axon growth while facilitating neovascularization and immune‐microenvironment remodeling; and iii) possessing suitable mechanical properties and stability to protect regenerated SCs and axons from further damage during nerve regeneration.^[^ ^28^ ^]^ Moreover, cumulative evidence has highlighted the advantages of electroactive materials in promoting cellular responses such as proliferation, migration, and differentiation for neural tissues.^[^ ^29^ ^]^ Graphene has been widely explored for biomedical applications because of its excellent electrical conductivity, superb mechanical property, and biocompatibility.^[^ ^30^ ^]^ For instance, graphene‐containing fibrous scaffolds can enhance the differentiation of neural cells and thereby facilitate the development of a 3D neuronal‐like network.^[^ ^31^ ^]^ For this purpose of enhancing peripheral nerve regeneration, we developed a conductive MF‐NGC with hierarchical fibers by integrating electrospinning and MEW printing techniques. MEW is a 3D printing technology that allows the direct writing of a variety of molten polymer filaments into fibers at low ranges of micrometers.^[^ ^32^, ^33^ ^]^

It is important to note that the materials used to fabricate the novel nerve conduits are either approved by the Food and Drug Administration (FDA) or clinically accepted to avoid any chronic inflammatory reaction post‐transplantation.^[^ ^34^ ^]^ For example, PCL, approved by the FDA for clinical use, has been widely demonstrated to be a biocompatible material for biomedical applications.^[^ ^35^ ^]^ The thermoplastic PCL polymer with a low melting point at ≈60 °C is a good candidate for MEW. The scanning electron microscope (SEM) micrographs (Figure 2d) showed that MEW can generate highly reproducible microfibers. Additionally, the micrographs illustrated that 3D stacking patterns can be produced by layer‐by‐layer assembly (Figure S1a, Supporting Information). By adjusting the fiber orientation, we obtained a multilayer mesh with orthogonally aligned PCL fibers (Figure S1b, Supporting Information) or parallelly aligned PCL fibers (Figure S1c, Supporting Information). The microfibers deposited by MEW are highly aligned and adjustable by controlling various parameters, including the applied voltage, gas pressure for dispensation, and translating speed (Figure S1d–f, Supporting Information). By optimizing printing parameters, we could generate microfibers with a diameter of 10.3 ± 2.6 µm, which was previously demonstrated to be effective at guiding cell alignment.^[^ ^19^ ^]^

To impart the multiscale NGCs with electroactive properties, we first prepared polycaprolactone‐graphene oxide (GO/PCL) composites in which GO was subsequently converted to reduced graphene oxide (rGO) by thermal treatment, thereby improving mechanical properties and restoring conductivity.^[^ ^36^ ^]^ In contrast to pristine graphene, graphene oxide (GO) displays significantly improved dispersion in solvents due to hydrogen bonding, endorsing high processability.^[^ ^37^ ^]^ Additionally, rGO produced through the reduction of GO is generally believed to be of lower toxicity than GO.^[^ ^38^ ^]^ As illustrated in Figure S2a (Supporting Information), the rGO/PCL composite was MEW‐printed onto PCL microfibers. The surface texture and morphology are similar between the PCL and PCL/rGO meshes, except that the PCL/rGO meshes have a blackish surface (Figure S2b, Supporting Information), which indicates the presence of rGO. More importantly, the addition of rGO significantly increased the fiber diameters of PCL up to hundreds of micrometers, as demonstrated by SEM (Figure S2c,d, Supporting Information). The dependence of the PCL/rGO microfiber diameters on these parameters was evaluated as shown in Figure S2e–g (Supporting Information), consistent with previous studies.^[^ ^17^ ^]^ In this study, a fiber diameter of 125 ± 12 µm (Figure 2e) was selected when the input voltage was kept at 3.5 kV, the solution flow rate was 20 µl min^−1^, the stage speed was 120 mm min^−1^, and the nozzle‐to‐substrate gap was 3 mm. Moreover, the MEW printing of PCL/rGO is more advantageous than the conventional FDM technique, as it protects the PCL microfibers at the bottom layers from the heating nozzle.

We used randomly organized PCL/collagen nanofibers as external shells of NGCs that provide a free exchange of nutrients and metabolic waste while preventing infiltration of the surrounding tissues. PCL/collagen nanofibers were fabricated through electrospinning, and the surface topographies of random nanofibers were verified using SEM (Figure S3, Supporting Information). The mean diameters of the random nanofibers were 574 ± 14 nm when the input voltage was kept at 12 kV, the solution flow rate was 40 µl min^−1^, and the nozzle‐to‐collector gap was 100 mm (Figure 2f). Therefore, the printed conduit consisted of hierarchical fibers with diameters ranging from 500 nm to 125 µm (Figure 2g). Using MEW printing, the NGCs were highly reproducible with oriented microfibers (Figure 2h,i). As shown in Figure 2j, the 3D‐printed MH‐NGC had a length of 15 mm (Figure 2j) and an exterior wall thickness of ≈500 µm (Figure 2k). SEM images of MF‐NGCs showed a fibrous sheath with random nanofibers and longitudinal microfibers (Figure 2l), which emulates the structural features of the natural epineurium and is considered beneficial for the formation of functional nerves. Furthermore, fibrous sheets consisting of PCL microfibers and rGO/PCL microfibers were densely packed into the lumen of the MF‐NGCs (Figure 2m,n). MF‐NGCs encapsulated with anisotropic, mechanically stable grid patterns (Figure 2o,p) exhibit a remarkable similarity to the architecture of nerve bundles, which is beneficial for neural growth and regeneration. Although the 3D printing of the outer tubular structure and the inner filler is a highly automated manufacturing process, the loading of the filler into the hollow conduit has to be performed manually for now. This can be challenging in a large‐scale production process where high consistency in loading is required to ensure product quality from a commercial perspective. Our future work will focus on optimizing the manufacturing process of MF‐NGCs to achieve high reproducibility and scalability.

### 2.2 Mechanical and Electrical Properties of MF‐NGCs

**Figure 3:**
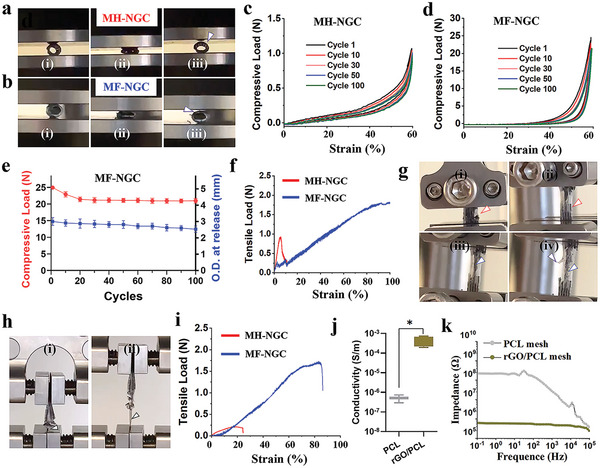
Mechanical and electrical properties of 3D printed MH‐NGCs and MF‐NGCs. a,b) Optical images of MH‐NGCs (a) and MF‐NGCs (b) under cyclic compression. i–iii) represent the deformation of MH‐NGCs and MF‐NGCs in three compression states: before the load, compression at 60% and after the load was removed (white arrow indicating the gap after compression release), respectively. c,d) Compressive load‐strain curves of MH‐NGCs (a) and MF‐NGCs (b) with different numbers of cycles. e) Maximum compressive load and outer diameter (O.D.) of MF‐NGCs with different numbers of cycles. f) The tensile load and deformation relationship of MH‐NGCs (red) and MF‐NGCs (blue) under uniaxial stretching. g) Optical images of MF‐NGCs under tensile stretching at different stages. Red, white, and blue arrows indicate rGO/PCL microfibers, random PCL nanofibers, and longitudinal PCL microfibers, respectively. h) MF‐NGCs in the suture pull‐out state. Black arrows indicate the nylon sutures. i) The tensile load and deformation relationship of MH‐NGCs (red) and MF‐NGCs (blue) under the suture pull‐put test. j) Electrical conductivity of the PCL and rGO/PCL meshes. k) Bode plots of EIS of the PCL and rGO/PCL meshes. Student's *t*‐test was used to analyze the data, *p < 0.05.

Previous NGCs often encountered mechanical failure when used in long‐term repairs, as implanted conduits would undergo repeated mechanical deformations.^[^ ^39^ ^]^ Therefore, the mechanical strength and stability of these NGCs are required to provide stable space for neural growth. The compression properties of the conduits are essential for long‐term shape maintenance in vivo. The mechanical durability of the MH‐NGC and MF‐NGC conduits was assessed by cyclic compression tests (**Figure** **3**a,b). The MH‐NGCs exhibited a good compression recovery capacity and structural integrity during compression (maximum 60% strain). The MH‐NGCs showed ≈96% of their initial compressive stress even after 100 compression cycles (Figure 3c). Similarly, the MF‐NGCs retained their mechanical strength, displaying ≈84.1% of the initial compressive stress after 100 cycles of compression without enduring severe structural damage (Figure 3d). The maximum stress of the MF‐NGCs was 20 times higher than that of the MH‐NGCs. The outer diameters (O.D.) of the conduits after compression release were calculated as shown in Figure 3e. The O.D. of the MF‐NGCs was initially 3 mm, which decreased to 2.53 mm after 100 cycles. A finite element analysis showed that the MF‐NGC displayed a significantly lower deformation than the MH‐NGC when subjected to a compression force of 5N. The mechanical strength effect is comparable to that of thicker fibers (200 microns). Similarly, in the tensile test, the tension in the MF‐NGCs was significantly higher than that in the MH‐NGCs during the entire test (Figure 3f). The elongations at the break of the MF‐NGCs were over 100%, showing good elasticity. The rGO/PCL microfibers first ruptured (indicated by red arrows) under tensile stretching, followed by cracking of random PCL nanofibers (indicated by white arrows) and longitudinal PCL microfibers (indicated by blue arrows) (Figure 3g).

Furthermore, NGCs need to have a high suture pull‐out strength to avoid failure during surgery. To simulate suture pull‐out, we performed a suture tensile test (Figure 3h). For the MH‐NGCs, it was difficult to hold the suture even when the loading force was only 0.25 N (Figure 3i). Thus, this sample group was unsuitable for implantation. The results show that MF‐NGCs can endure higher stress (≈1.7 N) at longer stretch distances (≈5 mm) than MH‐NGCs (Figure 2g). It should be noted that the incorporation (0.5 mg mL^−1^) of GO into PCL significantly improved the mechanical properties of the MF‐NGCs. The rGO/PCL meshes were significantly stiffer than the pure PCL meshes, which is attributed to the additional molecular interactions between rGO and the PCL polymer. These results suggested that our MF‐NGCs fulfilled the requirement of mechanical stability for implants, which is beneficial to protecting the regenerated SCs and axons from further damage during nerve regeneration.

The electrical properties of various conduits were investigated by electrical conductivity and electrochemical impedance spectroscopy (EIS). The rGO/PCL meshes showed a significantly higher conductivity ((4.3 ± 2.4) × 10^−4^ S m^−1^) than the pure PCL meshes ((5.1 ± 3.1) × 10^−7^ S cm^−1^) (Figure 3j), indicating the contribution of the conductive rGO portions in the composite rGO/PCL fibers. In addition, PCL had high impedances at all tested frequencies (0.1–10^5^ Hz), whereas the composite rGO/PCL meshes had lower impedances. For example, the impedance values of the PCL and rGO/PCL meshes at 1 Hz were 221 MΩ and 594 kΩ, respectively (Figure 3k). The results show that our MF‐NGCs containing the rGO/PCL meshes were electrically conductive, which may provide unconventional performance in potential peripheral nerve regeneration.

### 2.3 Neurite Outgrowth and SC Growth in Multiscale Fibrous Scaffolds

**Figure 4:**
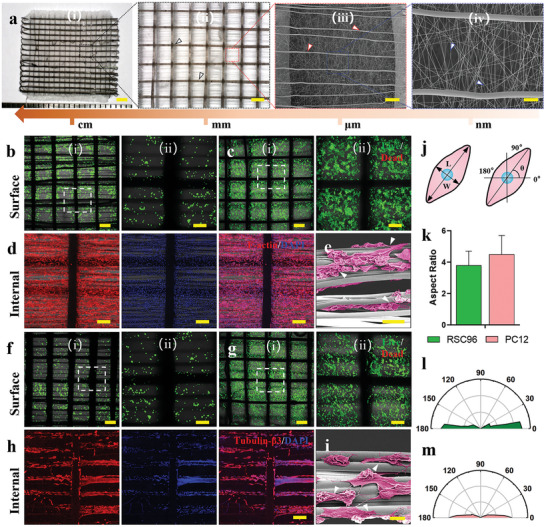
In vitro culture of RSC96 Schwann cells and PC12 neuronal cells on the flat multiscale fibrous scaffold. a) Morphological characterization of the multiscale fibrous scaffold. White arrows, blue arrows, and red arrows indicate the PCL/collagen nanofibers, PCL microfibers, and the rGO/PCL microfibers, respectively. Scale bars in Scale bars in i) 2 mm; ii) 500 µm; iii) 200 µm; iv) 100 µm. b,c) Live/dead staining of RSC96 cells on NGCs on day 1 (b) and day 7 (c). Scale bars in i) 500 µm; ii) 200 µm. d) *F*‐actin staining of RSC96 cells on NGCs on day 7. Scale bar: 200 µm. e) SEM image of RSC96 cells on NGCs on day 7. Scale bar: 100 µm. f,g) Live/dead staining of PC12 cells on NGCs on day 1 (f) and day 7 (g). Scale bars in i) 500 µm; ii) 200 µm. h) Tubulin beta III staining of PC12 cells on NGCs on day 7. Scale bar: 100 µm. i) SEM image of PC12 cells on NGCs on day 7. White arrows indicate neurite growth along with anisotropic microfibers. Scale bar: 100 µm. j) Schematic illustration of the orientation and aspect ratio of cells. k) Comparison of aspect ratios of RSC96 and PC12 cells on the NGC scaffold. l,m) Nightingale rose plots of orientation angle for RSC96 (l) and PC12 cells (m) on the NGC scaffold.

The biocompatibility of the NGC scaffold is very important for nerve tissue regeneration. Schwann cells (SCs) promote neurite growth in peripheral nerves by secreting neurotrophic molecules.^[^ ^40^ ^]^ RSC96 cells, as one of the most commonly used SCs, were seeded into flat multiscale fibrous scaffolds, that is, fibrous meshes consisting of random nanofibers on the surface and oriented microfibers in the interior as shown by SEM images (**Figure** **4**a). Cells on the flat surface were also observed as a control (Figure S5, Supporting Information). The cytocompatibility of flat multiscale fibrous scaffolds was confirmed by live/dead staining on day 1 and day 7 (Figure 4b,c). RSC96 cells were randomly distributed on the surface of the multiscale fibrous scaffold with high cell viability (greater than 95%). This is mainly attributed to the fact that the PCL and collagen components are noncytotoxic. However, most of the SCs presented an immature morphology with a round shape on day 1. The flat multiscale fibrous scaffolds with nanofibers and microfibers provided more cell adhesion sites for SCs. SCs grew significantly after 7 days of culture to cover the entire flat multiscale fibrous scaffolds, which indicated that the guidance of fibrous scaffolds promoted the proliferation of SCs. On day 7, most SCs residing within the scaffold showed a typical mature appearance with a spindle‐like shape, as shown by phalloidin staining (Figure 4d). SEM images showed a clear cell morphology of SCs along with the oriented microfibers, suggesting that the oriented microfibers guided the growth of SCs (Figure 4e). Our in vitro results indicated that the MF‐NGCs with oriented microfibers proved superior support for SC growth in nerve repair. This is in line with previous work showing that cells spread parallel to oriented microfibers, while the spread of SCs was disordered on random microfibers.^[^ ^19^ ^]^ The well‐distributed SCs in the MF‐NGCs provide a better microenvironment for axon extension during in vivo nerve regeneration.

The growth of neurons was studied by seeding PC12 cells into flat multiscale fibrous scaffolds. PC12 cells are a common neuron cell model with similar neural properties, including morphological differentiation.^[^ ^41^ ^]^ The viability and proliferation of PC12 cells on the scaffolds were similar to those of SCs (Figure 4f,g). We induced the neuronal differentiation of PC12 cells by using a nerve growth factor‐containing culture medium. The neurite outgrowth of PC12 cells after 7 days of culture was observed with immunofluorescence staining (Figure 4h) and SEM (Figure 4i). On the flat fibrous scaffolds, the PC12 cells grew freely, and most cells retained a small and round shape on day 1; however, the PC12 cells exhibited a dispersive and elongated shape on day 7 with the guidance of oriented microfibers. Contrarily, the RSC96 and PC12 cells exhibited a random distribution on the flat surface without topographical guidance (Figure S5, Supporting Information). In terms of axon length, the average length of neurites from PC12 cells significantly increased from 9.6 ± 2.7 µm on day 1 to 42.5 ± 12.8 µm on day 7, as measured using ImageJ software. The aspect ratio (Figure 4j,k) and orientation distribution (Figure 4l,m) of RSC96 and PC12 cells were quantitively measured based on the fluorescent images. The proportions of orientation within (−10°–10°) for RSC96 cells were more than 80% (Figure 4l), while the proportion of oriented PC12 cells was more than 90% (Figure 4m). These results indicated that the oriented microfibers within MF‐NGCs were essential in guiding neurite extension. This was consistent with previous studies that have demonstrated that oriented microfibers greatly enhance axon extension during sciatic nerve regeneration.^[^ ^35^ ^]^ Moreover, previous studies also revealed that the neurites of PC12 cells on the conductive fibrous meshes spread more vigorously along with the oriented microfibers than those on the nonconducting fibrous meshes.^[^ ^42^ ^]^ Therefore, it was reasonably inferred that NF‐NGCs filled with aligned conductive microfibers are an excellent choice for in vivo nerve regeneration. During in vivo nerve regeneration, oriented microfibers encapsulated with the conduit may guide SC growth to form the myelin sheath, which will further guide axonal extension.

### 2.4 Sciatic Nerve Regeneration after Transplantation of 3D Printed MF‐NGCs

#### 2.4.1 Histological Analysis of Regenerated Sciatic Nerves

**Figure 5:**
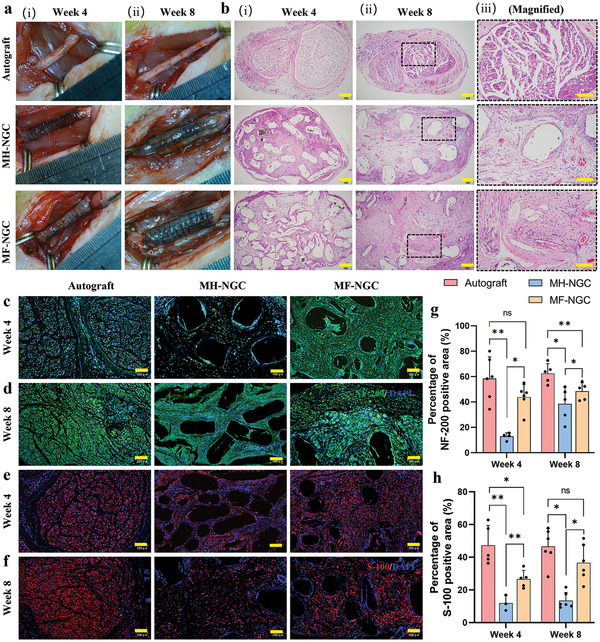
In vivo implantation of various NGCs and histological analysis of regenerated sciatic nerves. a) Photographs of the sciatic nerves regenerated by various NGCs at 4 and 8 weeks post‐implantation. b) H&E staining of the cross‐sections of regenerated nerve tissue after 4 and 8 weeks. Scale bars in i,ii) 200 µm; iii) 100 µm. c,d) Immunofluorescent staining of NF‐200 (green) and nuclei (blue) in the regenerated nerve fibers at 4 and 8 weeks post‐transplantation. e,f) Immunofluorescent staining of S100 (red) and nuclei (blue) in the regenerated nerve fibers at 4 and 8 weeks post‐transplantation. Scale bars in (c–f) 100 µm. g,h) Quantitative expression of NF‐200 (i) and S100 (j). Data are expressed as the mean values ± S.D. (n = 5). Student's *t*‐test was used to analyze the data, *p < 0.05, **p < 0.01, and “ns” indicates not significant.

Individual NGCs, including MH‐NGCs and MF‐NGCs, were fabricated at a length of 15 mm and implanted into rat sciatic nerve defect sites (length: 10 mm) of Sprague‒Dawley (SD) rats. Autografts were implanted as a positive control (**Figure** **5**a). All SD rats had successful surgery without any operative complications. At 4 and 8 weeks post‐transplantation, all NGCs were found to be present in all experimental animals without septic collection or inflammatory symptoms. In animals implanted with MH‐NGCs or MF‐NGCs, the nerves were found to be black, indicating that rGO/PCL fibers remained at the implanted site without a macroscopic inflammatory response. All NGCs were found to be partially degraded at week 8, which might lead to the release of rGO into the blood. Previous biodistribution studies (injected doses of 10 mg kg^−1^) have shown that intravenously injected GO was eliminated quickly from the blood and accumulated mainly in the lung and liver.^[^ ^43^ ^]^ After three months, the graphene sheets were completely eliminated by both renal and fecal elimination without signs of damage in major organs. Therefore, the rGO induces toxicity in a dose‐dependent pattern.^[^ ^44^ ^]^ In this study, there was only 0.5 mg rGO for an implanted NGC weighing 100 mg, which is relatively low for adult mice (adverse effects occur when intravenous injections exceed 10 mg kg^−1^ of body weight^[^ ^45^ ^]^), not to mention human bodies.

To estimate the regenerated nerve tissues, histological analysis was performed at both 4 and 8 weeks postoperatively. Cross‐sections of samples in the middle position were stained with hematoxylin‐eosin (HE) (Figure 5b) and toluidine blue (TB) (Figure S6, Supporting Information) to observe the overview of the regenerated nerve tissues. As seen in Figure 5b, regenerated nerve tissues extended into all conduits after 4 weeks of repair. No fibrous cell infiltration from the surrounding tissues to these conduits was observed in the histological images. The positive areas of HE staining in the MF‐NGC group were significantly more substantial than those in the MH‐NGC group at week 8, indicating higher degrees of cellular and tissue growth into defect sites. Similarly, the MF‐NGCs samples at the longitudinal sections showed higher degrees of tissue growth than the MH‐NGC samples at 8 weeks (Figure S7, Supporting Information). Moreover, the signal intensities were higher in the proximal and central regions than in the distal regions. This result confirmed that the topographic guidance cues of oriented microfibers within the conduits played a crucial role in guiding nerve regeneration.

**Figure 6:**
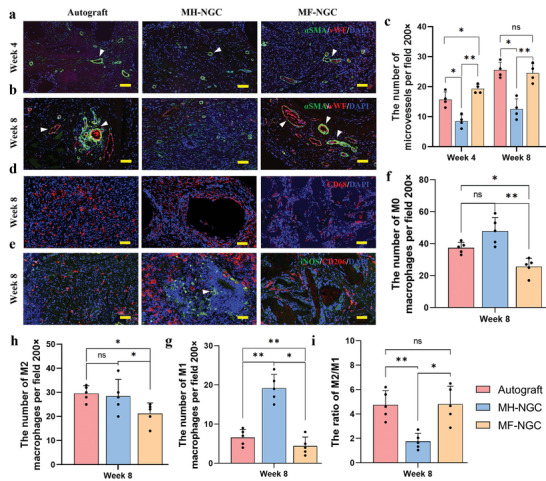
Neovascularization and macrophage polarization of regenerated nerve fibers. a,b) Immunofluorescent staining of vWF (red), *α*SMA (green), and nuclei (blue) showed the distribution of endothelial cells and vascular smooth muscle cells in the regenerated nerve fibers at 4 and 8 weeks post‐transplantation. White arrows point to the microvessels. c) Immunofluorescent staining of CD68 (red) and nuclei (blue) showed the distribution of M0 macrophages in the regenerated nerve fibers at 8 weeks post‐transplantation. d) Immunofluorescent staining of iNOS (green), CD206 (red), and nuclei (blue) showed the distribution of M1 and M2 macrophages in the regenerated nerve fibers at 8 weeks post‐transplantation. Scale bars in (c–f) 100 µm. e) Quantitative analysis of the functional capillaries. The white arrow indicates M1 macrophages. f–h) Quantitative analysis of the numbers of M0, M1, and M2 macrophages in different groups. i) Quantitative analysis of the ratio between M2/M1. Data are expressed as mean values ± S.D. (n = 5). Student's *t*‐test was used to analyze the data, *p < 0.05, **p < 0.01, and “ns” indicates not significant.

Regenerated sciatic nerves harvested at 4 and 8 weeks post‐transplantation were immuno‐stained at cross‐sections and analyzed to investigate axonal outgrowth and neuronal maturation (Figure 5c–f). A sparse positive neurofilament signal was observed at the cross‐sections of the nerves in the MH‐NGC group. At 4 weeks, the neurofilament‐positive area of MF‐NGCs (44.0% ± 9.8%) in the central region was significantly larger than that in the MH‐NGCs (13.9% ± 2.8%) and not substantially different from that of the autograft group (58.4% ± 17.2%) (Figure 5g). At week 8, neurofilament staining areas increased in all groups, and the differences among the samples increased. S‐100 staining and quantification of Schwann cell distribution in regenerated nerve cables through the NGCs showed results similar to neurofilament staining (**Figure** **6**h). For example, the MF‐NGC group showed fully matured myelinating Schwann cells with S‐100‐positive areas (26.7% ± 5.3%) in entire regions at 4 weeks, which was significantly higher than those in the MH‐NGCs (12.0% ± 4.7%) groups. Interestingly, the S‐100‐positive areas of the MF‐NGCs and the autograft groups were not significantly different, corresponding to 33.8% ± 9.6% and 50.3% ± 6.9%, respectively.

#### 2.4.2 Neovascularization and Macrophage Polarization of Regenerated Nerve Fibers

Microvessels play a vital role in nerve regeneration by providing the requisite material exchange and nutrition transportation for regenerated nerve tissues.^[^ ^46^ ^]^ We performed immunofluorescent staining for von Willebrand Factor (vWF) and smooth muscle actin (*α*‐SMA) to characterize the distribution of capillaries (Figure 6a,b). Newborn capillaries with red blood cells inside were found between the regenerated nerve tissues in all groups. Furthermore, microvessel density was statistically analyzed based on the immunohistochemical images of vWF in the whole sample after 4 and 8 weeks (Figure 6c). As expected, significantly higher microvessel density was found in the MF‐NGCs (week 8: 24.5 ± 3.1 per field 200×) than in the MH‐NGCs (week 8: 12.5 ± 3.4 per field 200×), indicating enhanced neovascularization in the multichannel conduit compared with the hollow conduit. Thus, NGCs filled with oriented microfibers can promote the restoration of defective peripheral nerves by inducing angiogenesis and neovascularization for nutrition exchange.

Furthermore, the number of macrophages significantly increased in the nerve bridges of MF‐NGCs at weeks 4 and 8 compared to MH‐NGCs. Such a notable difference implied that oriented microfibers filled within conduits were more effective at promoting cell migration for recruitment. We further evaluated the polarization of macrophages in the nerve regeneration bridges by immunofluorescent staining for selected polarization markers (iNOS and CD206) (Figure 6d,e). The total numbers of pan‐macrophages (M0), pro‐inflammatory macrophages (M1), and pro‐healing macrophages (M2) were identified by immunofluorescent staining for CD68, iNOS, and CD206, respectively (Figure 6f,g). Statistical analysis showed that the ratio of M2/M1 (4.8 ± 1.5) in the MF‐NGC group was significantly higher than that (1.8 ± 0.7) in the MH‐NGC group at week 8 (Figure 6i). The results showed that oriented microfibers enabled the rapid recruitment of macrophages and promoted the M2 transition.

Such findings were consistent with previous studies that oriented microfibers promoted macrophage polarization to the M2 phenotype.^[^ ^46^ ^]^ For example, Want et al. demonstrated that macrophages were primarily located on the surface of oriented microfibers and exhibited an M2 phenotype.^[^ ^47^ ^]^ It should be noted that the regulation heavily depended on the dimensions of topological cues. For example, 20‐µm oriented microfibers were more favorable toward M2 macrophage polarization than 50‐µm microfibers.^[^ ^48^ ^]^ Therefore, we developed intraluminal guidance structures with oriented microfibers (10.3 ± 2.6 µm) between subcellular (5 µm) and cellular (30 µm) scales that encouraged M2 macrophage polarization and neurite outgrowth. The incorporation of intraluminal structures within a hollow nerve conduit has proven to be a simple yet effective strategy for guiding nerve regeneration while preventing the invasion of surrounding connective tissue^[^ ^49^ ^]^; moreover, our results indicate that conduits filled with conductive‐oriented microfibers facilitate the recruitment of macrophages and their transition to the pro‐regenerative M2 phenotype in a timely manner, which in turn enhances the migration, proliferation, and myelination of SCs and axon extension to promote functional regeneration of damaged nerves.

#### 2.4.3 Promotion of Myelinated Fiber Regeneration In Vivo

**Figure 7:**
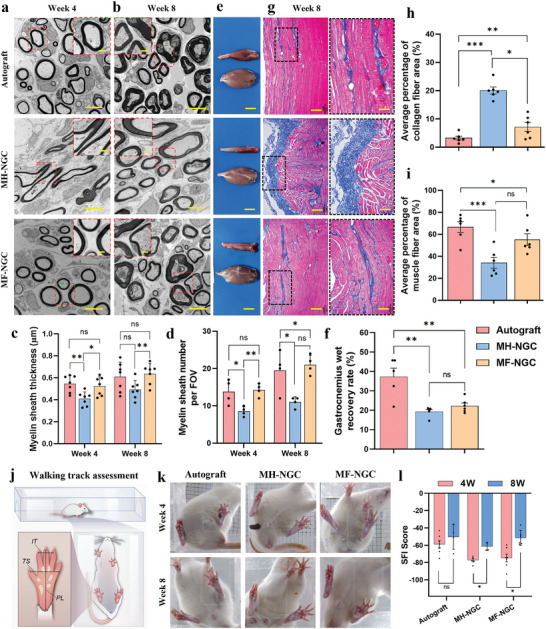
Functional reconstruction of the regenerated sciatic nerve after surgery. a,b) Representative TEM images of the regenerated nerve fibers at 4 and 8 weeks post‐transplantation. Scale bars, 5 µm (subset: 1 µm). c,d) Quantitative analyses of c) nerve myelin sheath thickness and d) myelinated fiber numbers. e) Representative images of gastrocnemius muscles of both hindlimbs harvested at 8 weeks post‐transplantation. Scale bars, 10 mm. f) GWWRR of targeted gastrocnemius muscles (operated side/contralateral normal side). g) Representative images of Masson's trichrome staining of cross‐sectional gastrocnemius muscle. Blue staining indicates collagen fiber deposition and muscle fibrosis. Scale bars, 200 µm (subset: 100 µm). h) Percentage of collagen fiber area of the targeted gastrocnemius muscles. i) Percentage of muscle fiber area of the targeted gastrocnemius muscles. j) Schematic illustration of walking track assessment. k) Walking footprint of the SD rats at 4 and 8 weeks post‐transplantation. l) SFI values of the sciatic nerves calculated from a walking footprint. Data are expressed as mean values ± S.D. (n = 5). Student's *t*‐test was used to analyze the data, *p < 0.05, **p < 0.01, ***p < 0.005, and “ns” indicates not significant.

The remyelination of axons is essential for the proper transmission of signals in the PNS.^[^ ^50^ ^]^ However, the myelin sheaths of axons that are naturally remyelinated after PNIs are often thin. A large axon as well as a thick myelin sheath indicate successful regeneration of the sciatic nerve.^[^ ^51^ ^]^ Accordingly, myelin sheath thickness and numbers were investigated using transmission electron microscopy (TEM) images of cross‐sections of the central region of regenerated nerves (**Figure** **7**a,b). In the autograft and MF‐NGC groups, most regenerated axons were surrounded by thick, clear, and electron‐dense myelin sheaths. In contrast, the MH‐NGC groups showed thin and loose myelin sheaths with vacuolar‐like defects, which are typical features of demyelination. Myelin sheath thickness in the MF‐NGC group (522 ± 85 nm) was significantly higher than that in the MH‐NGC group (409 ± 62 nm) and was not substantially different from that of the autograft group (544 ± 75 nm) at 4 weeks (Figure 7c). At week 8, myelin sheath thickness increased in all groups, and the differences among the samples increased (Figure 7c). In addition, myelin sheath numbers were significantly higher in the MF‐NGCs (19 ± 2 per FOV) than in the MH‐NGC groups (9 ± 2 per FOV), which were slightly higher than that in the autograft group but showed no significant difference (16 ± 3 per FOV) (Figure 7d).

#### 2.4.4 Gastrocnemius Muscle Recovery

After nerve transection, the gastrocnemius atrophied but reinnervated after nerve regeneration.^[^ ^52^ ^]^ The gastrocnemius was isolated from rat hind limbs 8 weeks post‐transplantation (Figure 7e). The left (experimental) gastrocnemius muscle in all groups showed muscle shrinkage, that is, atrophy at 8 weeks. Regeneration of muscles after innervation was evaluated by measuring the weights of the left and right gastrocnemius muscles in each group (Figure 7f). The gastrocnemius wet weight recovery ratio (GWWRR) in the reinnervated muscles of the MF‐NGC groups (22.3% ± 3.5%) was slightly higher than that of the MH‐NGC groups (19.2% ± 2.6%). In addition, the GWWRR values of both the MH‐NGCs and MF‐NGC groups were significantly smaller than that of the autograft group (37.2% ± 10.1%). Masson's trichrome staining was performed on transverse sections of the gastrocnemius to reveal collagen fiber deposition and muscle fibrosis, a common feature of muscle atrophy (Figure 7g). Morphometric analysis showed degradation and fragmentation of muscle fibers in the MH‐NGC groups (Figure 7g). The autograft group (57 ± 5 µm) and MF‐NGCs (44 ± 8 µm) had significantly thicker muscle fibers than MH‐NGCs (19 ± 6 µm) at 8 weeks. To quantitatively assess the degree of muscle fibrosis, we further calculated the average percentages of collagen and muscle fiber area in gastrocnemius muscles using ImageJ (Figure 7h,i). The collagen fiber area was significantly smaller in the MF‐NGC groups (7.1% ± 4.0%) than in the MH‐NGC groups (20.0% ± 3.0%) and was slightly higher than that in the autograft group (3.2% ± 1.6%). This finding suggests that MF‐NGCs could significantly alleviate muscle atrophy induced by axon truncation following their implantation.

#### 2.4.5 Leg Functional Recovery

We conducted a walking track analysis to study pathology and potential nerve injury treatment and evaluate the functional recovery of sciatic nerves post‐NGC implantation (Figure 7j). As previously reported, a decrease in the function of the sciatic nerves usually results in a narrowing of both toe spread and intermediate toe spread.^[^ ^52^ ^]^ Footprint analyses at 4 and 8 weeks revealed wider toe spread and intermediate toe spread in the autograft and MF‐NGC groups than in the MH‐NGC group (Figure 7k). The sciatic functional index (SFI) dramatically decreased for rats in all groups immediately after the complete transection of the sciatic nerve. Four weeks after implantation, the SFIs of MH‐NGCs (−77.4 ± 4.2) were lower than those of autografts (−58.9 ± 10.8) and MF‐NGCs (−74.7 ± 11.5) (Figure 7l). At 8 weeks, the MH‐NGC group still exhibited a significantly lower SFI than the other groups. In particular, the MF‐NGC group showed a greatly improved (higher) SFI (−51.5 ± 8.6) than the MH‐NGC group (−61.4.9 ± 4.6), which had a similar SFI to that of the autograft group (−50.4 ± 14.3) (Figure 7l). Therefore, our results demonstrated that the MF‐NGCs supported nerve regeneration at the histological level, comparable to autografts.

## 3 Conclusion

With the aim of developing an effective scaffold for PNI regeneration, we fabricated conductive MF‐NGCs by integrating MEW and electrospinning techniques. We successfully demonstrated that the conductive MF‐NGCs displayed multiple functions at various scales, including: i) at the nanoscale, the electrospun random PCL/collagen nanofibers (diameter: ≈500 nm) consist of an outer layer of MF‐NGCs, providing excellent permeability for nutrient diffusion and waste removal while impeding the infiltration of fibrous scar tissues; ii) at the microscale, the MEW‐printed oriented PCL microfibers (diameter: ≈10 µm) consist of an internal layer of MF‐NGCs, providing anisotropic guidance for neuronal growth, the rapid recruitment of vascular cells, and the pro‐regenerative M2 differentiation of macrophages; and iii) at the mesoscale, the MEW‐printed rGO/PCL microfibers (diameter: ≈125 µm) consist of an intermediate layer of MF‐NGCs, providing the desired mechanical stability and electroactive properties to support neural growth. Importantly, animal studies with a 10 mm peripheral defect model successfully demonstrated that MF‐NGCs can greatly facilitate neural regrowth, myelination, and functional regeneration of nerve tissues and muscle tissues. Our conductive MF‐NGC opens up new avenues for the treatment of PNIs. In the next step, we hope to incorporate neural stem cells into MF‐NGCs to better mimic the in vivo environment for peripheral nerve regeneration.

## 4 Experimental Section

### Synthesis of rGO/PCL Composites

The rGO/PCL composite was prepared by adapting to the literature.^[^ ^53^ ^]^ Briefly, 2 g of PCL pellets (Mn = 8 000, Sigma‒Aldrich) were dissolved in acetone (10 mL), and the stock GO solution (Aladdin, Shanghai) was added to the PCL solution to obtain 0.5% (w/w) GO to PCL. The solutions were stirred overnight and sonicated for 2 h to allow the dispersion of GO. The solvent was evaporated, and the nanocomposite was heated to 150 °C to thermally reduce GO to rGO. In this study, an rGO/PCL composite that contains 0.5 w/w% of rGO was used.

### Electrospinning of PCL/Collagen Nanofibers

Separate solutions were made of 10% w/v PCL or 10% w/v collagen (Solarbio, Beijing) dissolved in 1,1,1,3,3,3‐hexafluoro‐2‐propanol (HFIP; purity 99.5%) (Aladdin, Shanghai). PCL/collagen solution consisting of 10% w/v PCL/collagen (100:75) in HFIP was stirred at room temperature overnight, drawn into a syringe (22G), and pumped vertically into a drum collector. The electrospinning parameters included a voltage of 12 kV, a distance of 100 mm between the needle tip and the collector, and a flow rate of 40 µl per minute.

### Melt Electrowriting of PCL and rGO/PCL Microfibers

A custom‐built MEW device with a high‐voltage source, a controller, and a gas‐pressured feeding system was used to manufacture the microfibers. For PCL microfibers, PCL pellets were loaded into a stainless steel syringe with a 24 G blunt‐end stainless steel needle. The syringe was heated to 80 °C by a clamp heater for half an hour, resulting in a homogeneous polymer melt. Before extruding the polymer melt out from the needle, the syringe was connected to a filtered gas supply pump through the tubing. The pressure applied to the syringe was controlled to be 5 kPa. A high voltage of 4 kV was connected to a stainless steel plate collector while the spinneret was grounded. The distance between the needle and the collector was 4 mm. The collector was mounted on an automated stage, where the movement was controlled by editing the G‐code within motion control software. All the MEW experiments were performed under ambient conditions (25 °C, 30% relative humidity). For rGO/PCL microfibers, 0.5% rGO/PCL was heated to 100 °C, and extruded under a pressure of 10 kPa, and voltage of 3.5 V.

### Fabrication of MF‐NGCs

For the fabrication of MF‐NGCs, the first orthogonal PCL microfibers were printed on a rotating mandrel (diameter: 3 mm) by MEW, followed by MEW printing of rGO/PCL microfibers and electrospinning of PCL/collagen nanofibers. The multilayer conduits were carefully removed from the mandrel after printing to obtain a hollow conduit, that is, MH‐NGC. Second, a multilayer fibrous sheet was printed using alternate MEW printing of PCL microfibers and rGO/PCL microfibers. Finally, the fibrous sheet was rolled up and filled into the lumen of an MH‐NGC to obtain an MF‐NGC.

### Morphology Characterization

The morphology of the 3D‐printed structures was examined using SEM. To evaluate the internal structures of the MH‐NGCs and MF‐NGCs, cross‐sections were cut for surface analysis. The samples were freeze‐dried, sputter‐coated with gold (thickness, 20 nm), and imaged with a Zeiss Auriga SEM system at 10 kV. The average fiber diameter was determined by analyzing randomly selected fibers (at least 50) of the SEM micrographs using ImageJ software.

### Mechanical Testing

The mechanical properties of various NGCs were examined by cyclic compression tests and tension tests at room temperature using a mechanical test instrument (Bose ElectroForce 3200, Bose Corp.). The printed NGC scaffolds were stored in a vacuum‐sealed container before mechanical testing to avoid the influence of moisture on chemical stability. Before the measurement, NGCs 15 mm in length were fully immersed in PBS (HyClone, USA) for 30 mins. In the compression test, a pair of parallel plates was used to apply a compressive load perpendicular to the longitudinal axis of the wet conduit at its full length. Each testing group contained five replicates (n = 5), and the strain rate was 1 mm min^−1^ to test the maximum anti‐compression capacity at a strain of 50%. Resilience after compression was measured with a fast speed of 5 mm min^−1^ and a strain of 50%, and each measurement was repeated for 100 cycles without preloading. To characterize the suturing strength during implantation, 6‐0 sutures were used to sew one end of the NGCs, while a machine chuck fixed the other end of the NGCs. Then, a strain rate of 0.5 mm min^−1^ was applied.

### Finite Element Mechanics Analysis

The finite element mechanics analysis on the NGC scaffolds was performed using ANSYS 15.0. Computer‐aided design (CAD) software (SolidWorks 2018, Dassault Systemes) was used to generate 3D models of the MH‐NGC and MF‐NGC. As PCL/collagen nanofibers (diameter: 500 nm) and PCL microfibers (diameter: 10 m) were mechanically weaker than rGO/PCL microfibers (diameter: 125 m; fiber gap: 550 m), they were excluded from the 3D models in order to reduce computation time. As previously reported,^[^ ^53^, ^54^, ^55^ ^]^ the rGO/PCL microfibers possessed Young's modulus of 520 MPa, a Poisson ratio of 0.442, and a density of 1.14 g cm^−3^. With the bottom part of the scaffold fixed, a compression force of 5 N was applied to the top of the scaffold. As control groups, MH‐NGCs with thick fibers at 200 m and narrower fiber gaps at 350 m were simulated.

### EIS and Electrical Conductivity Measurement

For EIS measurement, the flat multiscale fibrous scaffold samples (n = 5) were preincubated in Dulbecco's phosphate‐buffered saline (DPBS) and then placed between two parallel gold‐coated glass electrodes. An alternative sinusoidal potential of 10 mV was applied in a frequency range of 1–10^5^ Hz using a computer‐assisted electrochemical device (CHI660D, Chenhua Ltd., China). To measure the conductivity of the flat multiscale fibrous scaffolds, a four‐point probe approach was employed at room temperature. Pure PCL fibrous sheets were used as a control. Sheet resistance was measured at a scan rate of 50 mV s^−1^ by linear scanning voltammetry, and the conductivity of each flat sample was calculated according to the literature.^[^ ^22^ ^]^

### In Vitro PC12 and RSC96 Cell Culture

Murine pheochromocytoma (PC12) neuronal cells and rat Schwann cells (RSC‐96) were purchased from Cell Resource Center, Beijing, and used to assess the cytocompatibility of the MF‐NGCs in vitro. PC12 and RSC96 cells were cultured in a culture medium consisting of Dulbecco's modified Eagle's medium (Invitrogen Gibco., USA), 10% fetal bovine serum (Invitrogen Gibco., USA), and 1% streptomycin‐penicillin (50 U mL^−1^, Invitrogen Gibco., USA). Flat multiscale fibrous scaffold samples were sterilized by incubation in 75% ethanol for 2 h, followed by washing with sterile DPBS and exposure to ultraviolet light for 4 h. Next, PC12 and RSC96 cells were seeded onto the MF‐NGC samples at a density of 1.0 × 10^4^ cells cm^−2^. After the culture of PC12 and RSC96 cells, cell viabilities were assessed using a Calcein‐AM/PI Double Staining Kit (Dojindo Molecular Technologies). On day 7, samples were fixed with paraformaldehyde for 30 min, washed with DPBS, and incubated in permeabilization/blocking solution (0.1% v/v Triton X‐100, 5% w/v BSA, and 2% v/v goat serum in DPBS) at 4 °C for 2 h. Next, PC12 cell‐laden samples were stained with a primary anti‐beta III tubulin antibody (1:100, Mouse monoclonal antibody, Abcam) and a goat anti‐mouse IgG1 Alexa 488 secondary antibody (1:200, Abcam). RSC96 cell‐laden samples were stained with phalloidin for the cytoskeleton. All samples were incubated in 4ʹ,6‐diamidino‐2‐phenylindole (DAPI) solution (1:1000 in DPBS) before visualization with fluorescence microscopy (FV3000, Olympus). In this study, the quantitative neurite outgrowth from PC12 cells was measured based on high‐magnification fluorescent images using NeuronJ, a plugin for ImageJ. The average length of neurites per cell was determined by dividing the total outgrowth by the number of cells (at least 20 cells).

### Animal Model and Transplantation

Adult male SD rats (8 weeks old, weight: 200–250 g) were used as a sciatic nerve defect model as previously described.^[^ ^56^ ^]^ Animal study procedures were approved by the Institutional Animal Care and Use Committee of Zhonghong Boyuan Biotechnology Co., Ltd, Jiangxi, China (AP#2021110801). Animals provided free access to food and water were randomly divided into three experimental groups: the Autograft group (represented as Autograft) (n = 12), the MH‐NGC scaffold graft group (defined as MH‐NGCs) (n = 12), and the MF‐NGC scaffold group (described as MF‐NGCs) (n = 12). Surgical procedures were performed under anesthesia using 2% (w/v) pentobarbital sodium solution (40 mg kg^−1^). Under aseptic conditions, the skin and subcutaneous muscle layers in the left hip joint were incised to expose the left sciatic nerve. Nerve fibers were transected at 5 mm proximal to the nerve bifurcation and removed, leaving a 10 mm gap. Next, each end of the transected sciatic nerve was inserted into a 15 mm long individual NGC (n = 12 per group) and then tied to the epineuria sheath using 9‐0 nylon. The muscle, subcutaneous layer, and overlying skin were sutured using 5‐0 silk. Following resuscitation from anesthesia, rats were housed in their cages as usual. At 4 and 8 weeks, rats were sacrificed using carbon dioxide.

### Histological Assessment of the Regenerated Nerve

The regenerated nerve was dissected immediately after the animal behavior evaluation at weeks 4 and 8 post‐surgery. Hematoxylin and eosin (H&E), TB, and TEM were used to observe the cross‐sectional morphology of regenerated nerves. For HE staining, all nerve samples were fixed with 4% paraformaldehyde, embedded in paraffin, and cut into sections of 5 µm with a microtome (Leica). For TB staining, the nerve conduits were cut into 1 µm transverse sections and stained with TB solution (Servicebio, China). For TEM, all nerve samples were fixed with 2.5% glutaraldehyde, post‐fixed with 1% osmium tetroxide, dehydrated, embedded in a Poly/Bed 812 Embedding Kit (Polysciences Inc., Warrington), and cut into ultrathin sections of 70 nm. These ultrathin sections were stained with uranyl acetate and lead citrate and then examined via TEM (Titan, China) at a voltage of 80 kV. The number and thickness of the myelin sheath were quantitatively analyzed using ImageJ software.

The sections were stained with anti‐neurofilament 200 (NF‐200) (1:400, rabbit polyclonal antibody, Abcam, ab204893) and anti‐S100 (1:400, mouse monoclonal antibody, Abcam, ab7852) primary antibodies to assess nerve myelin protein and nerve axon protein of the regenerated nerve, respectively. Similarly, the sections were stained with anti‐von Willebrand Factor (1:200, rabbit polyclonal antibody, Abcam, ab6994) and anti‐alpha smooth muscle actin (1:100, mouse monoclonal antibody, Abcam, ab7817) to assess the vascularization of the regenerated nerves. The sections were stained with anti‐CD68 (M0, pan‐macrophage) (1:100, mouse monoclonal antibody, Abcam, ab31630), anti‐iNOS (M1, pro‐inflammatory) (1:70, rabbit polyclonal antibody, Abcam, ab15323), and anti‐Mannose Receptor (i.e., CD206, M2, anti‐inflammatory) (1:100, rabbit polyclonal antibody, Abcam, ab64693) to assess macrophage polarization within the regenerated nerves. The following secondary antibodies were used: goat anti‐mouse IgG1 Alexa 488 (1:200, Abcam, ab150117) and goat anti‐rabbit IgG Alexa 594 (1:200, Abcam, ab150116). The nuclei were counterstained with DAPI. Images were observed with an immunofluorescence microscope (FV3000, Olympus). The microvessel density and the ratio of M2/M1 were measured by ImageJ software.

### Gastrocnemius Wet Weight and Histological Evaluation

After the rats were sacrificed, the gastrocnemius muscles on both sides of the rats were harvested at 4 and 8 weeks after implantation. The harvested muscles (n = 6) were washed in PBS and weighed using an electronic scale. After that, the gastrocnemius muscles were fixed in 4% formaldehyde overnight, incubated in sucrose solutions of different concentrations (10%, 15%, and 30%) for 2 days at 4 °C, embedded in optimal cutting temperature compound, and cut into transverse sections with a thickness of 10 µm. After deparaffinization, the muscle sections were stained using a Masson's Trichrome Stain Kit (Solarbio, China). The muscle tissue images were captured under a microscope, and five random fields were selected for each group. The degree of muscle fibrosis was analyzed using ImageJ software to calculate the average percentage of collagen fiber area in gastrocnemius muscles.

### Animal Behavior Test

A walking trajectory analysis was performed to measure the functional recovery of rat sciatic nerves at 4 and 8 weeks post‐transplantation as adapted from the literature.^[^ ^48^ ^]^ Rats in each group (n = 6) were placed into a transparent‐bottom plastic box large enough for them to move freely. A high‐definition Webcam was suspended beneath the plastic box to record the rats' movements without interference. The toe spread (TS, first and fifth toe), intermediary toe spread (ITS, second and fourth toe), and paw length (PL, the third toe and heel) of the bilateral hind paws were measured for both the uninjured (N; “normal”) and injured (E; “experimental”) sides by ImageJ software. These values were then utilized to calculate the SFI as follows:

(1) $$\mathrm{SFI}=-38.3\left(\mathrm{EPL}-\mathrm{NPL}\right)/\mathrm{NPL}+109.5\left(\mathrm{ETS}-\mathrm{NTS}\right)/\mathrm{NTS}$$

Generally, the SFI ranges from −100 to 0, where −100 indicates complete neurological dysfunction and 0 indicates good repair.^[^ ^48^ ^]^

### Statistical Analysis

All the experimental statistical data were analyzed from at least three parallel samples and expressed as mean ± standard deviation. The differences between experimental groups were analyzed by Student's *t*‐test, and one‐way ANOVA followed by Tukey's HSD post‐hoc testing with GraphPad Prism software (version 9.0) for Windows. P values (*p < 0.05, **p < 0.01, and ***p < 0.005) were considered to be statistically significant.

## Conflict of Interest

The authors declare no conflict of interest.

## Supporting information

Supporting Information

## Data Availability

The data that support the findings of this study are available in the supplementary material of this article.
