# Supplementary material for: 3D Printed Conductive Multiscale Nerve Guidance Conduit with Hierarchical Fibers for Peripheral Nerve Regeneration
Source: Adv Sci (Weinh). 2023 Feb 17;10(12):2205744. doi: 10.1002/advs.202205744 (PMC10131803; doi:10.1002/advs.202205744)
Supplement: Supplementary file 1 — Supporting Information [file ADVS-10-2205744-s001.pdf]

ADVANCED SCIENCE
Open Access

Supporting Information

for *Adv. Sci.*, DOI 10.1002/advs.202205744

3D Printed Conductive Multiscale Nerve Guidance Conduit with Hierarchical Fibers for Peripheral Nerve Regeneration

Yongcong Fang, Chengjin Wang, Zibo Liu, Jeonghoon Ko, Li Chen, Ting Zhang, Zhuo Xiong, Lei Zhang* and Wei Sun**

3D printed conductive multiscale nerve guidance conduit with hierarchical fibers for peripheral nerve regeneration

Yongcong Fang[1,2,3], *Chengjin Wang*[1,2,3], *Zibo Liu*[1,2,3], *Jeonghoon Ko*[1,2,3], *Li Chen*[1,2,3], *Ting Zhang*[1,2,3], *Zhuo Xiong*[1,2,3]*, *Lei Zhang*[1,2,3]*, *Wei Sun*[1,2,3,4]*

[1]Biomanufacturing Center, Department of Mechanical Engineering, Tsinghua University, Beijing 100084, P. R. China

[2]Biomanufacturing and Rapid Forming Technology Key Laboratory of Beijing, Beijing 100084, P. R. China

[3] "Biomanufacturing and Engineering Living Systems" Innovation International Talents Base (111 Base), Beijing 100084, P.R. China

[4]Department of Mechanical Engineering, Drexel University, Philadelphia, PA 19104, USA

E-mail:

xiongzhuo@tsinghua.edu.cn;

stoneszhang@mail.tsinghua.edu.cn;

weisun@mail.tsinghua.edu.cn

Supplementary info.

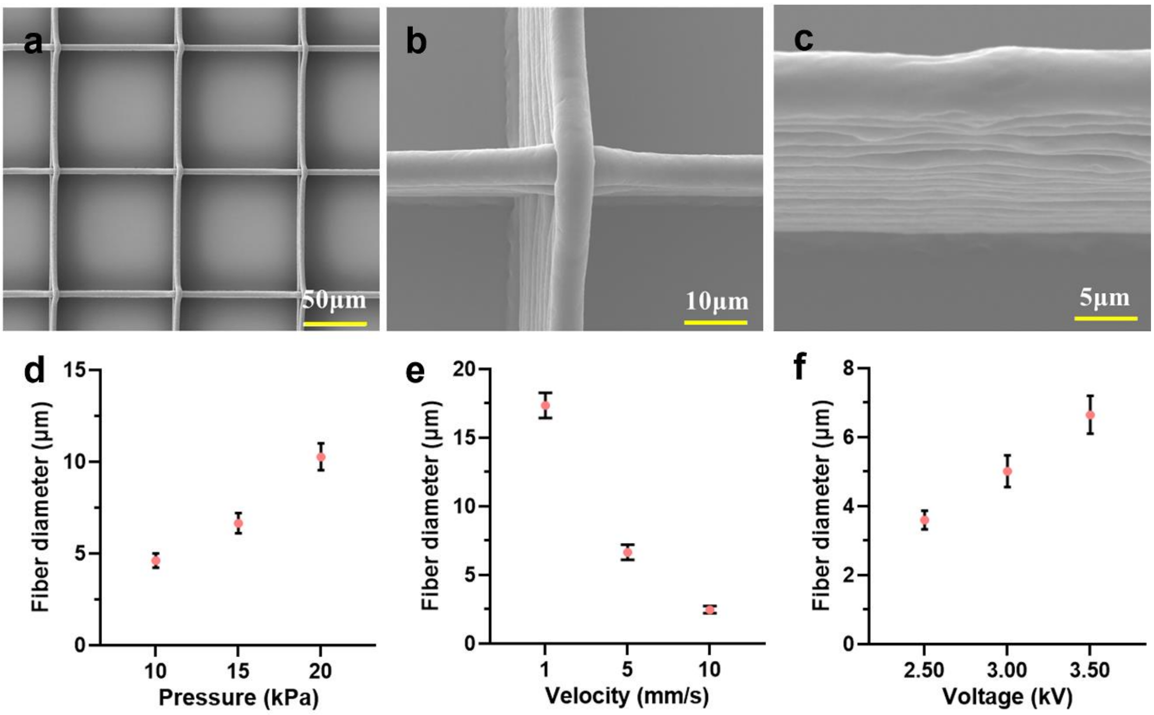

Figure S1. MEW printing of PCL microfibers and the effect of printing parameters on fiber diameters. (a) The grid pattern printed by MEW. (b) Magnification of a multilayer mesh with orthogonally aligned PCL fibers. (c) Magnification of a multilayer mesh with parallelly aligned PCL fibers. Scale bars in (a) 500 µm; (b) 10 µm; (c) 5 µm. (d-f) The effect of printing parameters including gas pressure for dispensation, translating velocity, and applied voltage on fiber diameters.

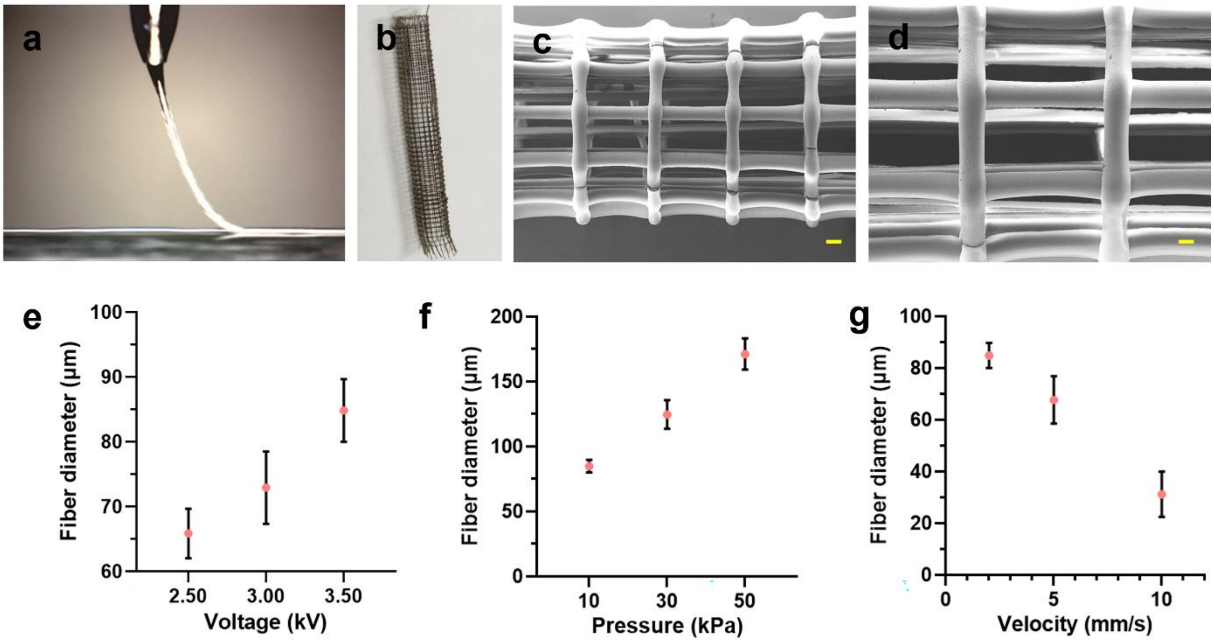

Figure S2. MEW printing of rGO/PCL microfibers and the effect of printing parameters

on fiber diameters. (a) Optical image of the MEW printing process of PCL microfibers. (b) Optical image of the conduit that consists of orthogonally aligned rGO/PCL fibers. The rGO renders the conduit a dark blackish color. (c) SEM image of the conduit by MEW printing of rGO/PCL fibers. (d) A magnified view of (c). Scale bars in (c) 200 µm; (d) 100 µm. (e-f) The effect of printing parameters, including applied voltage, gas pressure for dispensation, and translating velocity on fiber diameters.

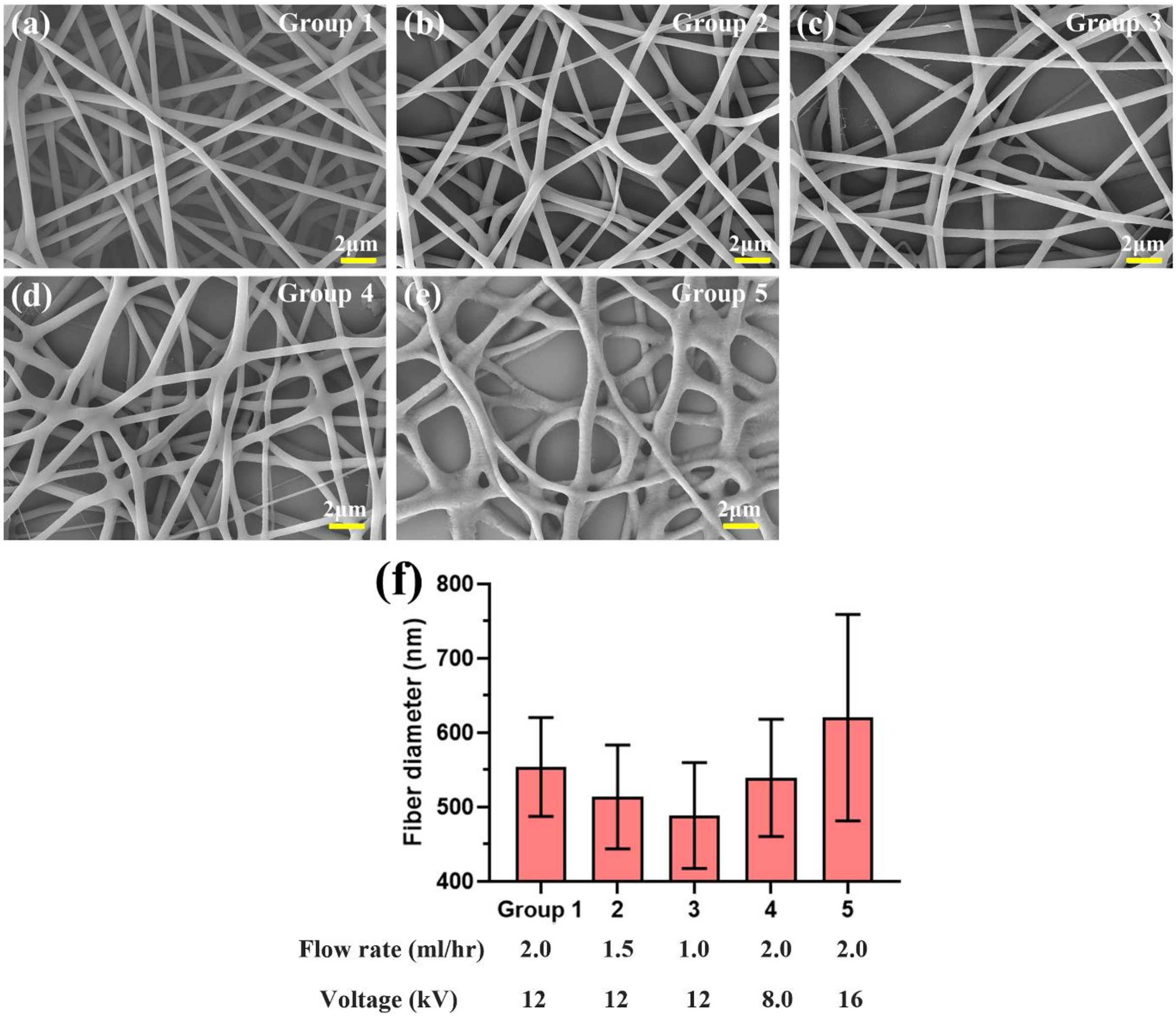

Figure S3. Electrospinning of PCL/collagen nanofibers and the effect of parameters on fiber diameters. (a-e) SEM image of electrospun PCL/collagen nanofibers at various parameters. Scale bars in (a-e) 2 µm. (f) The diameter distribution of PCL/collagen nanofibers at various parameters.

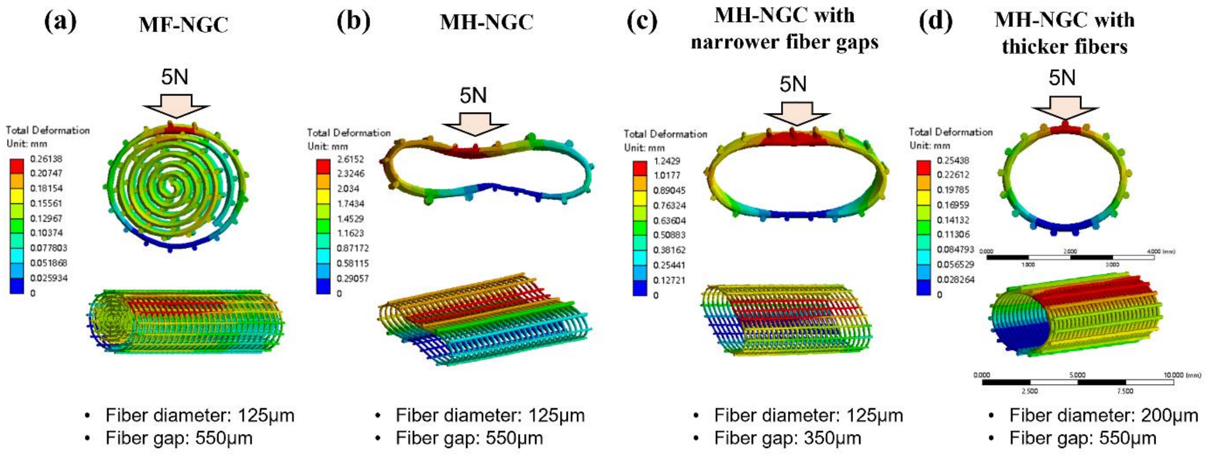

Figure S4. Finite element analysis of the deformation of NGC scaffolds to compression. (a) MF-NGC with fiber diameter at 125 μm and fiber gap at 550 μm; (b) MH-NGC with fiber diameter at 125 μm and fiber gap at 550 μm; (C) MH-NGC with fiber diameter at 125 μm and fiber gap at 350 μm; (d) MF-NGC with fiber diameter at 200 μm and fiber gap at 550 μm.

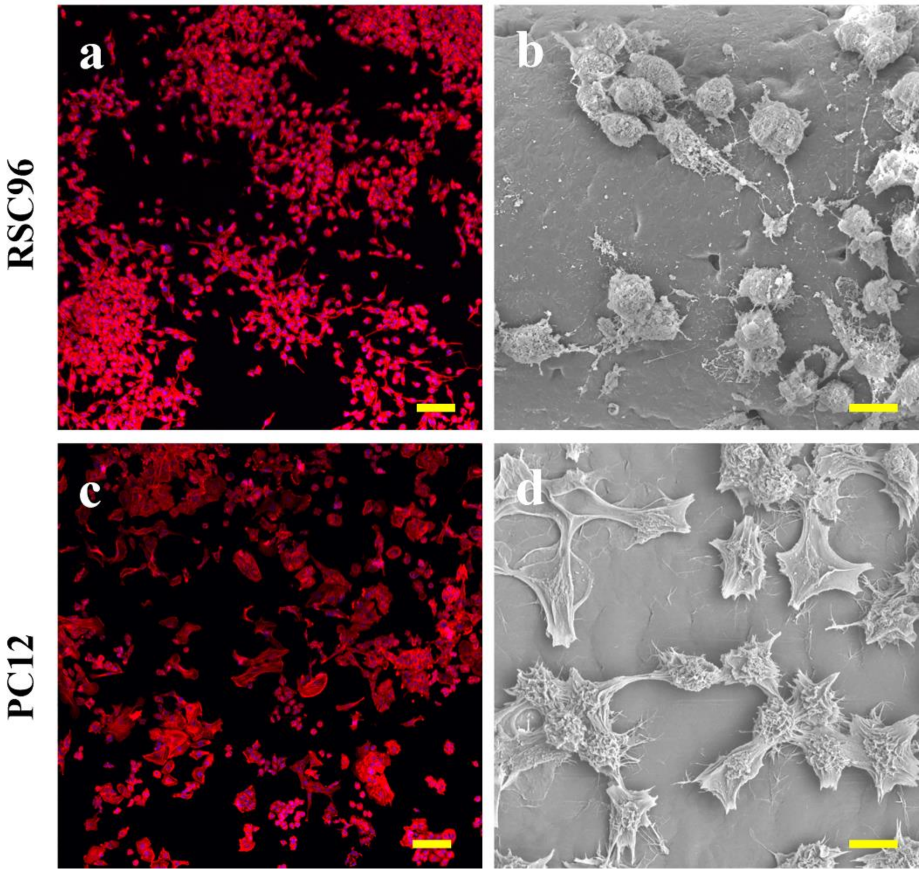

Figure S5. In vitro culture of RSC96 Schwann cells and PC12 neuronal cells on a flat polymer surface. (a,c) F-actin staining of RSC96 and PC12 cells on day 7. (b,d) SEM image of RSC96 and PC12 cells on day 7. Scale bars in (a,c) 100 µm; (b,d) 10 µm.

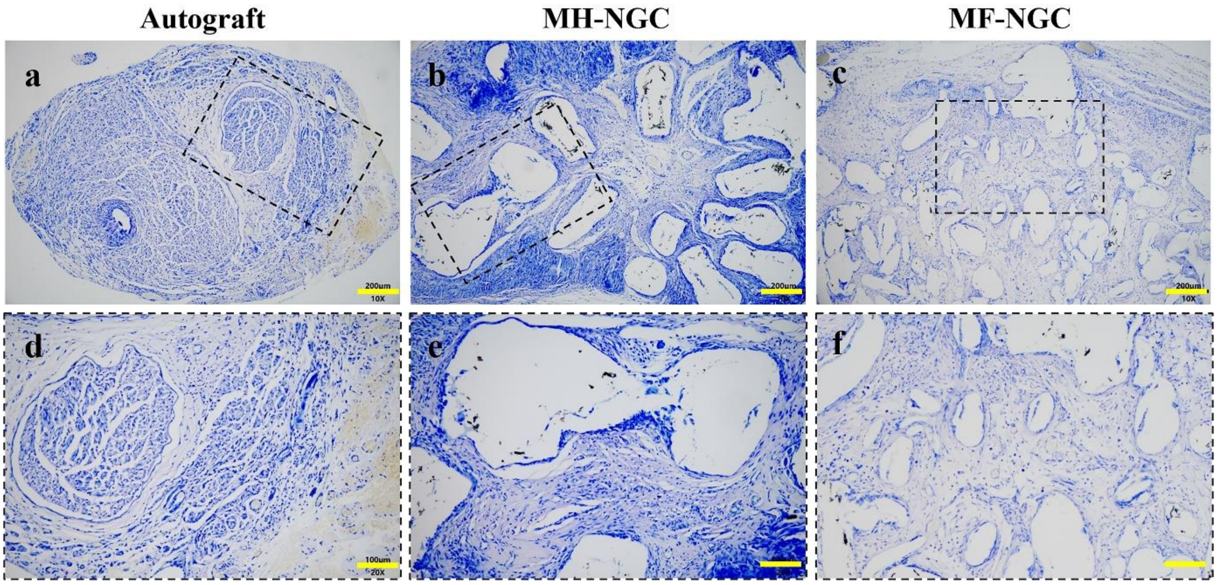

Figure S6. Representative toluidine blue-stained images of the regenerated nerve fibers at

8 weeks post-transplantation. (a,d) Autograft group; (b,e) MH-NGC group; (c,f) MF-NGC group. (d-f) Magnified views in (a-c), respectively. Scale bars in (a-c) 200 μm; (d-f) 100 μm.

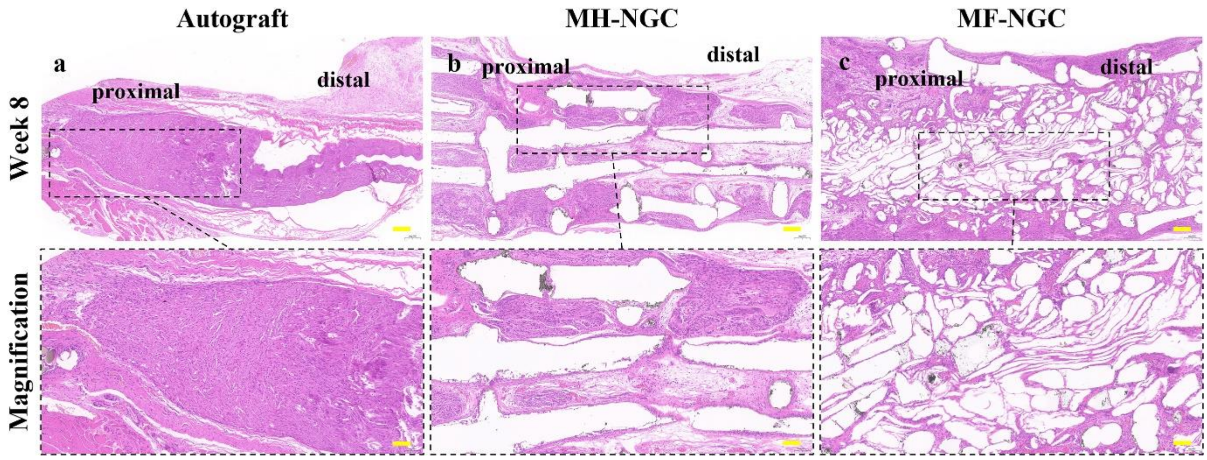

Figure S7. Representative H&E staining images of longitudinal sections of regenerated nerve tissue after 8 weeks. Scale bars in (a-c) 200 μm (subset: 100 μm).
